# Oviposition Dynamics and Niche Utilization in Two Sympatric *Drosophila* Species

**DOI:** 10.1007/s10886-025-01576-4

**Published:** 2025-02-04

**Authors:** Rolando D. Moreira-Soto, Bill S. Hansson, Markus Knaden

**Affiliations:** 1 Department of Evolutionary Neuroethology, Max-Planck Institute for Chemical Ecology, Jena, Germany; 2 Universidad de Costa Rica, Centro de Investigación en Enfermedades Tropicales, Facultad de Microbiología, San José, Costa Rica

**Keywords:** *Drosophila*, Oviposition behavior, Niche utilization, Interspecies interaction, Coexistence, Aggregation

## Abstract

Many *Drosophila* species coexist by sharing their feeding and breeding sites, which may influence their oviposition choices in an interspecies social context. Whether and where to lay eggs is a crucial decision for female flies as it influences the success of their offspring, by minimizing the risk of predation, competition, or cannibalism. Significant gaps exist in our understanding of *Drosophila* oviposition dynamics in co-occurring species. Here we tested oviposition strategies of *Drosophila melanogaster* and its close relative *Drosophila simulans* under different conditions, to assess whether a single female would prefer to oviposit separately or together with another female, be it a conspecific or not. We find that ovipositing females, regardless whether they are conspecifics or not, prefer to oviposit at the same site. This might suggest that the flies regard the benefits of sharing oviposition sites as higher than the potential risks of competition or cannibalism. The willingness to share oviposition sites was lower when the nutritional value of the medium was increased by adding yeast, and was lost when flies were allowed to lay the eggs consecutively, instead of being tested together. The latter might be explained by our additional finding that females become attracted by the presence of other females on oviposition substrates and that this attraction is partly driven by visual cues. Ovipositing in groups might facilitate intra- and interspecific social feeding of same age offspring, as well as enrichment of microbes. However, this cooperation dynamic might change if another female’s offspring is already present, as it might be perceived as danger of competition or cannibalism.

**Supplementary Information:**

The online version contains supplementary material available at 10.1007/s10886-025-01576-4.

## Introduction

Many *Drosophila* species in nature utilize the same feeding and breeding sites and are drawn to the same fermenting fruit baits, presenting a clear example of coexistence (Budnik & Brncic 1974). *Drosophila melanogaster* and *Drosophila simulans* are part of what is called the cosmopolitan guild, a group of species that share similar ecological niches (Atkinson & Shorrocks 1977). It has been suggested that the population dynamics of coexisting *Drosophila* species are likely influenced, at least in part, by interspecies competition (Budnik & Brncic 1974). While resource competition in animals can lead to exclusion, studies show that coexistence can be stable in certain environments or conditions, with one species not always completely eliminating the other, as shared resources could lead to cooperative behaviors (Ayala 1970; Budnik & Brncic 1974; Miller 1964). Competition between species is not limited to resource exploitation; it can also involve interference, where the presence of one species alters the efficiency or behavior of the other (Budnik & Brncic 1974).

The cooperation and competition dynamics are evident in insects, where density-dependent signals, such as pheromones or other chemosensory cues, can influence reproductive and oviposition behavior (Dumenil et al. 2016; Ferveur 2005; Tungadi et al. 2022). In *Drosophila*, females have been shown to adjust their egg-laying strategies based on the presence and density of other *Drosophila* eggs or larvae on a substrate (Bailly et al. 2023; Moreira-Soto et al. 2024). In *Aedes aegypti* mosquitoes it is known that females aggregate more often than expected in one oviposition site over others, when equal choices are offered (Costa-da-Silva et al. 2024). The ultimate decision to deposit eggs by a female insect demands a complex integration of sensory inputs, from finding a breeding site to assessing its quality (Costa-da-Silva et al. 2024). Reports in *Drosophila* show that oviposition engages multiple sensory modalities, including vision, olfaction, proprioception, and taste (Dweck et al. 2013; Liu et al. 2017). While sensory neurons on the ovipositor play a key role in the final decision to lay an egg (Chess & Ringo 1985; Takamura & Fuyama 1980), other appendages, such as the proboscis, wings, and legs, also contain taste receptors with sex-specific responses that may influence this decision-making process (Chyb 2004; Markow & O’Grady 2008; Meunier et al. 2000; Stocker 1994).

The selection of oviposition sites plays a critical role in the survival and fitness of an animal’s offspring (Richmond & Gerking 1978). In *Drosophila* flies, which have immobile egg and pupal stages, and larvae with limited mobility, the choice of oviposition site is paramount, as this means that the immature stages cannot move to better substrates (Markow 2015; Richmond & Gerking 1978). Therefore, poor site selection increases vulnerability to predation, and reduces chances for larval survival, directly influencing the reproductive success (Durisko et al. 2014; Refsnider & Janzen 2010). Also, laying eggs communally can lead to challenges such as resource competition, restricted growth, and even cannibalism when resources are exhausted (Bailly et al. 2023; Etienne et al. 2002; Narasimha et al. 2019; Wertheim et al. 2002). The significance of this choice is highlighted by numerous studies showing that females are highly selective about where they lay their eggs and can delay oviposition until they find an optimal substrate (Azanchi et al. 2013; Fanara et al. 2023; Joseph et al. 2009; Schwartz et al. 2012; Yang et al. 2008). Thus, natural selection is expected to exert strong pressures on behaviors related to oviposition, particularly under conditions of resource scarcity and high competition (Markow 2015).

In terms of joint egg laying, it has been suggested that females are more likely to be attracted to cues linked to favorable species, genotypes, and population densities (Beltramí et al. 2012). For example, in *Drosophila suzukii*, studies have shown that females are deterred from ovipositing when they detect the presence of *D. melanogaster* larvae. Additionally, when they detect allospecific egg cues, they avoid certain species while showing no preference for others (Kidera & Takahashi 2020). Similar results have been observed in *D. melanogaster*, where females were attracted to oviposit near conspecific eggs and some allospecifics but showed no preference for others (Moreira-Soto et al. 2024). Larvae of different species have been shown to interact, often with negative consequences for some species: *D. simulans* had a negative effect on *Drosophila rufa* and *Drosophila immigrans*, but its own fitness was unaffected (Takahashi et al. 2005).

In the present study, we investigated the oviposition preference of *D. melanogaster* and *D. simulans* under different conditions, given multiple oviposition sites, to assess whether they would prefer to oviposit separately or together with another female, and if the latter, whether it matters if the other female is a conspecific or not. We also tested aggregation at potential oviposition sites to determine whether flies follow conspecifics or heterospecifics using visual and/or olfactory cues when selecting where to lay their eggs. By examining these behaviors under various conditions, we expand the knowledge on the factors that affect coexistence in shared environments. As these species coexist in nature, they provide a valuable model for studying the complexities of niche utilization and species coexistence. The interplay between competition and cooperation in these sympatric species highlights the intricate behavioral and ecological mechanisms that allow for coexistence in shared environments, offering insights into how species balance resource use and reproductive strategies in natural systems.

## Results

### Oviposition Site Preference of *D. melanogaster* and *D. simulans* in 4 Choice Assays

**Fig. 1:**
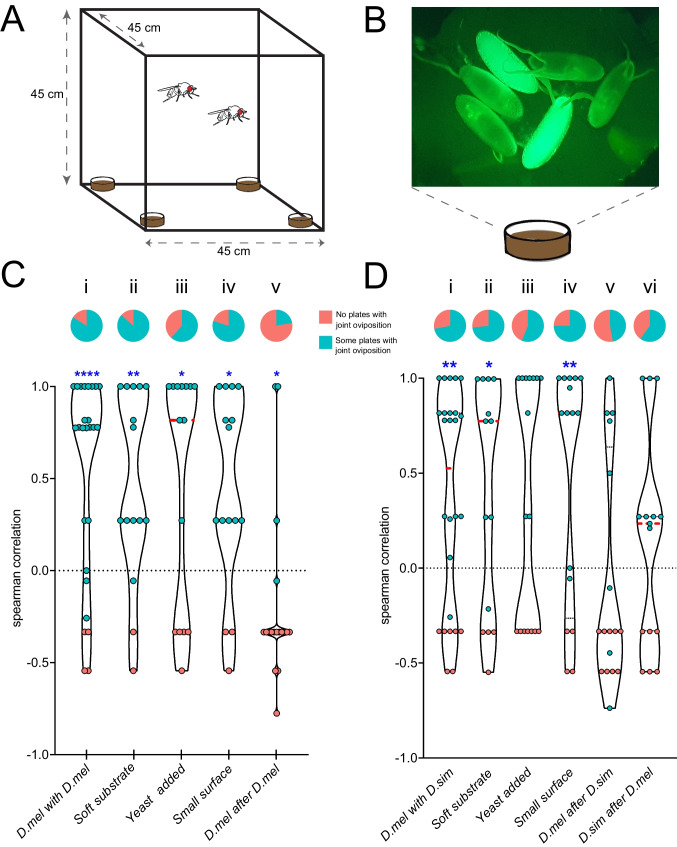
(**A**) Visual representation of the 4-choice oviposition setup. (**B**) Example image of fluorescent eggs from *D. melanogaster* (RRID:BDSC_4534) and non-fluorescent eggs from *D. melanogaster*. (**C**) Oviposition assays in *D. melanogaster* (RRID:BDSC_4534 and wild type for i-iv; 2 wild type flies for v) tested under different conditions (i-v). (**D**) Oviposition assays of *D. simulans* with *D. melanogaster* (RRID:BDSC_4534 for i-iv; wild type for v-vi) tested under different conditions (i-vi). Top graph, proportion of assays that had no plates with joint oviposition (red) or at least one plate with joint oviposition (green; see Table S1); bottom graph, distribution of spearman correlation values for all individual assays; dashed red lines indicate the median and dashed black lines indicate the quartiles. High values depict more joint, low values depict more separate oviposition. Blue stars indicate statistical difference from zero (*n* = 15–26, Wilcoxon signed-rank test, *P < 0.05; **P < 0,01; ***P < 0.001, ****P < 0.0001). For raw data of (**C**) and (**D**) see Supplemental Data 1

We examined the oviposition preference for 4 equal oviposition substrates (Fig. 1A), to determine if *D. melanogaster* females would prefer to oviposit together with females of *D. simulans* or conspecifics. In order to later distinguish *D. melanogaster* from *D. simulans* eggs, we used a *D. melanogaster* fly line (RRID:BDSC_4534) expressing GFP ubiquitously under control of Act5C (Fig. 1B). The cuticular chemicals of this fly line are indeed similar to that of wild type *D. melanogaster,* as the wild type samples group close to the mutant fly line in the UMAP (Fig. S1). Further, we found that only 4.6% of the chemical compounds have difference in abundance between this fly line and the wild type flies (Tukey’s test for multiple comparisons of means (P < 0.05)). We found no differences in the abundances of the male specific compounds: (Z)−11-Octadecen-1-yl acetate (cVA), (Z)−7-Tricosene and (Z)−7-Pentacosene, as well as female-specific compounds: (Z,Z)−7,11-Pentacosadiene, (Z,Z)−7,11-Heptacosadiene, and (Z,Z)−7,11-Nonacosadiene, reported for *D. melanogaster* (Khallaf et al. 2021). Given this, the results from this fly line in oviposition experiments should be transferable to wild type flies.

When first testing the oviposition choice of two synchronously tested females in a four-choice assay (Fig. 1A) we found a significant correlation of the number of eggs on a given oviposition site of the 2 flies tested, regardless, whether they were conspecific (Fig. 1C_i_) or allospecific (Fig. 1D_i_). Obviously, the flies preferentially oviposit on the same sites, even if they have several oviposition sites available. *Drosophila* larvae are known to cooperate while digging themselves into a substrate (Durisko et al. 2014). We therefore asked whether the females’ preference to oviposit together is driven by this, and is reduced when cooperative digging is not needed. Thus, we repeated the assay using 4 plates with softer agar. Despite the potentially reduced need for larval cooperation the female flies still oviposited together, be it with conspecifics (Fig. 1C_ii_) or allospecifics (Fig. 1D_ii_). It is possible that substrate hardness, and consequently the need for larval cooperation to soften the substrate, do not primarily govern the females' preference for joint oviposition sites; however, further tests are needed to confirm this.

*Drosophila* larvae are known to mainly consume microbes growing on the oviposition substrate and it was shown that flies during oviposition transfer microbes to the substrate (Bakula 1969; Stamps et al. 2012). One could, hence, speculate that female flies by laying eggs together increase the chance of their offspring for a nutritionally rich substrate. In reverse, a nutritionally rich substrate could reduce the motivation for cooperative oviposition. Indeed, when we added yeast to the substrate generally more flies decided to lay eggs, with 88.7% of the assays resulting in egg laying, compared to an average of 58.9% in all assays (Table S2), suggesting that yeast improved the substrate quality. Furthermore, at least the willingness of *D. melanogaster* to share the niche with *D. simulans* disappeared (Fig. 1D_iii_), while the cooperative oviposition of conspecific *D. melanogaster* females remained unchanged (Fig. 1C_iii_).

We next tested the flies’ preferences in a situation of increased competition, by reducing the surface they had to oviposit on. Under these conditions only 36.9% of the experiments resulted in egg laying, suggesting that this reduction indeed is sensed by the females (Fig. 1C_iv_). However, in the cases where the flies did oviposit, we observed that despite the potentially increased competition the preference to oviposit together remained, both for conspecific and allospecific flies (Fig. 1C_iv_ and D_iv_).

As *Drosophila* larvae have been reported to feed on *Drosophila* eggs (Narasimha et al. 2019), we finally asked whether a fly would avoid places where older eggs (that might present future danger for cannibalism) are already present. We therefore let the two tested flies lay their eggs successively. For this, the first fly was allowed to oviposit alone, and only after it was removed the second fly was tested. When testing allospecific combinations both *D. melanogaster* and *D. simulans* females lost their preference for joint oviposition, when the eggs/larvae of the first fly were already present (Fig. 1D_v__+vi_). Interestingly, when *D. melanogaster* females were tested after conspecific females had already oviposited, the flies even significantly avoided the already occupied oviposition plates (Fig. 1C_v_).

### Oviposition site aggregation of mated females of *D. melanogaster* and *D. simulans*

**Fig. 2:**
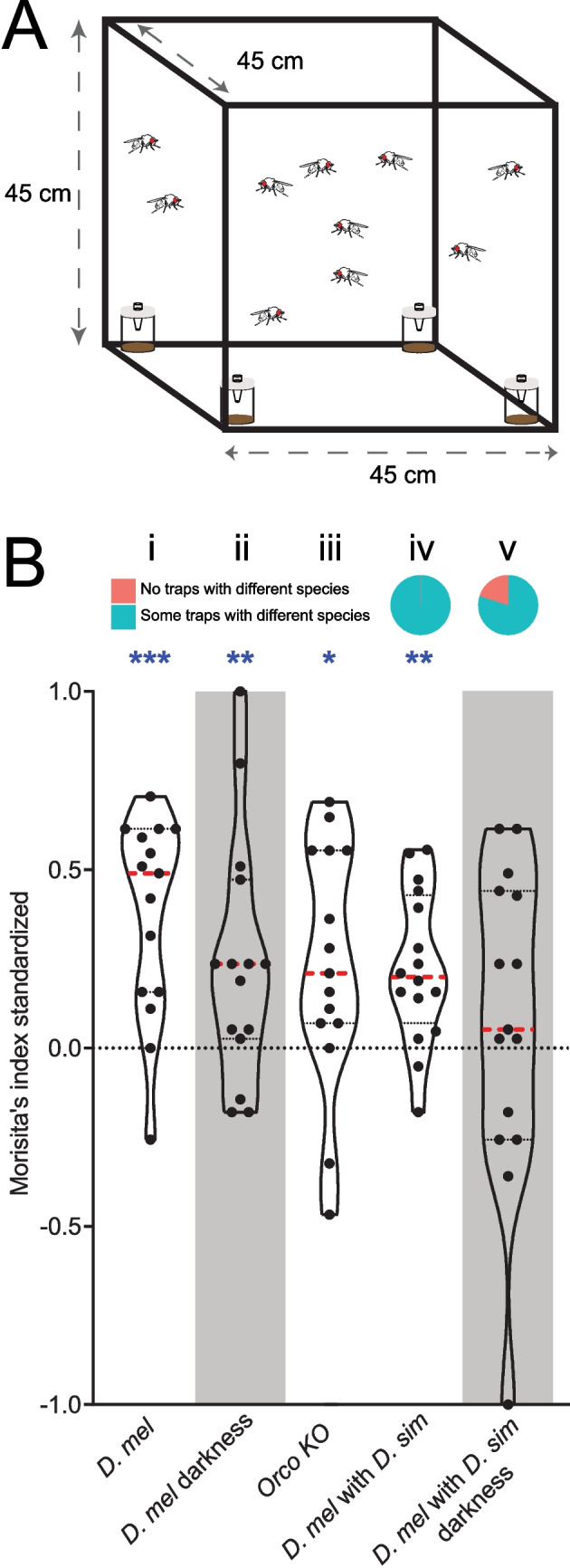
(**A**) Visual representation of the 4-choice trap assay. (**B**) Trap choices under different conditions (i-v). Top, proportion of assays in which traps only had one of the 2 species (red) or with at least one trap with both species (green); bottom, distribution of the Morisita’s index of aggregation for the different assays. Dashed red lines indicate the median and dashed black lines indicate the quartiles. High values depict more aggregation in traps. Gray areas indicate the assays performed in darkness. Blue stars indicate statistical difference from zero (*n* = 15–16, Wilcoxon signed-rank test, *P < 0.05; **P < 0,01; ***P < 0.001, ****P < 0.0001). For raw data of (B) see Supplemental Data 1

To investigate whether females are influenced by the presence or absence of other females when selecting an oviposition site we used a similar setup as before. However, instead of oviposition plates, we introduced four traps (i.e. small containers filled with fly food that could be entered but not exited easily), using a pipette tip as the entry point (Fig. 2A). This setup allows us to test whether flies follow each other into potential oviposition sites, in contrast to the oviposition assay, where they might visit food plates at different times, guided by other cues.

Instead of testing two individual flies, we also increased the number of tested flies to 10 (i.e. either 10 *D. melanogaster* females, or 5 *D. melanogaster* and 5 *D. simulans* females) per assay. Again, *D. melanogaster* females did not randomly end up in the different traps, but rather aggregated in some traps (resulting in a positive Morista’s index in Fig. 2B), regardless whether they were tested with conspecifics (Fig. 2B_i_) or with *D. simulans* females (Fig. 2B_iv_). The conspecific aggregation remained in the absence of visual cues (i.e. when tested in darkness; Fig. 2B_ii_) and when partly anosmic flies (i.e. flies with a non-functional olfactory co-receptor Orco; Fig. 2B_iii_) were tested. Obviously, several sensory modalities are involved in this conspecific aggregation. Interestingly, however, *D. melanogaster*’s aggregation with *D. simulans* disappeared in darkness, suggesting that *D. melanogaster* females are not attracted by the different smell (Fig. S1) of *D. simulans* females and their tendency to join *D. simulans* females is mainly driven by visual cues (Fig. 2_v_).

## Discussion

Drosophilid flies are known to have species-specific chemical cues, and these cues provide information for intra- and inter-specific communication (Antony et al. 1985; Bartelt et al. 1985; Ferveur 2005; Khallaf et al. 2021; Laturney & Billeter 2016; Moreira-Soto et al. 2024; Tungadi et al. 2023; Yew et al. 2009). Here, we investigate the niche overlap of the two closely related drosophilids *D. melanogaster* and *D. simulans* that are known to inhabit the same habitats in nature (Atkinson & Shorrocks 1977). Like other species, these two species exhibit species specific chemical profiles both as adults and on their eggs (Fig. S1; (Moreira-Soto et al. 2024)). We investigate the female flies’ preference to cluster their eggs with those of other females and the dependency of this behavior on whether the other females are conspecifics or instead belong to the closely related species. Our results indicate that ovipositing females, whether from the same species or not, tend to cluster their eggs on the same oviposition sites. However, the preference attenuated when the nutritional value of the medium increased (added yeast), and this preference was lost when females were allowed to lay eggs sequentially rather than simultaneously, thus encountering previously laid eggs or larvae (Fig. 1C,D).

These results suggest that the advantages of cooperation with same age larvae outweigh the risks of competition both within and between both species. This could potentially lead to larvae forming social foraging groups and by that enhancing their ability to burrow into difficult to penetrate substrate (Durisko et al. 2014). The group foraging strategy offers several benefits: first, it enables larvae to penetrate the fruit more quickly, where temperature and humidity are more stable than on the surface, and where they may be better shielded from parasitoids; second, burrowing by larvae can help to break down and soften the food, making it easier to consume; and finally, the larval digging may also mix the food substrate, potentially preventing the growth of competing molds and encouraging the development of beneficial yeast species (Bakula 1969; Durisko et al. 2014; Rohlfs 2005a, 2005b; Stamps et al. 2012).

Joint oviposition potentially presents several other advantages. It is known that ovipositing females inoculate the food with beneficial yeast (Stamps et al. 2012). In addition, the flies’ association with conspecifics or other species could help them find suitable food more quickly than they could on their own (Sarin & Dukas 2009). Studies on social behavior in larvae have shown that there is aggregation of larvae even when the food is very soft and easy to penetrate, suggesting that larvae may benefit from copying the choices of others, which could give them information to find a good substrate (Durisko & Dukas 2013). Also, Orengo and Prevosti (1994) showed that larval survival of *Drosophila subobscura* and *Drosophila pseudoobscura* was improved by the presence of allospecific larvae (i.e. *D. pseudoobscura* or *D. subobscura*), indicating a positive interaction.

On the other hand, reports show that aggregation comes with costs, when larvae compete for food, which might limit their growth and adult body size, and could eventually lead to cannibalism when resources are depleted (Allee 1927; Courchamp et al. 1999; Etienne et al. 2002; Narasimha et al. 2019; Vijendravarma et al. 2013; Wertheim et al. 2002). In accordance with this, competition between *D. melanogaster* and *D. simulans* when grown together for several generations, resulted in *D. melanogaster* displacing *D. simulans* (Hedrick 1972; Hedrick & King 1996). While in our experiments, despite of these potential risks, the flies decided to cluster their eggs, this effect disappeared when we let the flies oviposit successively (Fig. 1B_v_ and C_v_). In a recent study we showed that the presence of eggs attracts ovipositing females (Moreira-Soto et al. 2024). However, the lack of attraction reported here for successively ovipositing females may be due to the 24 h delay between the tests of the two flies, which may have resulted in the first larvae already hatching before the second fly was being tested. It has been reported that first instar larvae can predate on conspecifics (Ahmad et al. 2015; Vijendravarma et al. 2013), meaning that young larvae might represent a risk for the offspring of the ovipositing female. Furthermore, interspecific interactions can have asymmetric effects, where one species is competitively superior, negatively impacting the other, while remaining unaffected by the presence of the other species (Takahashi et al. 2005).

Do flies actually follow each other to potential oviposition sites? When we tested groups of gravid females in a trap assay where they could decide to enter traps together or not, again we found joint choices regardless, whether flies were conspecifics or not (Fig. 2_i_ and _iv_). While joint choices remained even in the absence of visual or the reduction of olfactory cues when we tested the aggregation within *D. melanogaster* (Fig. 2_iii__+iv_), these flies lost their attraction to *D. simulans*, when tested in darkness (Fig. 2_v_). It is already established that oviposition in *Drosophila melanogaster* is affected by social cues (Sarin & Dukas 2009), with naïve flies, after seeing other flies, increasing their own oogenesis (Bailly et al. 2023) and obviously even mimicking the flies’ oviposition choices (Battesti et al. 2012). On the other hand, *D. melanogaster* mated females are known to use aggregation pheromones that strongly attract other flies (Bartelt et al. 1985). Therefore, the social context driving oviposition choice probably is detected by multiple sensory modalities. Our data suggest that the joint oviposition is partly driven by the flies following each other, which again seems to be governed by several sensory modalities.

One example of an aggregation pheromone in *Drosophila melanogaster* is 11-cis-vaccenyl acetate (cVA), a chemical produced exclusively by males and transferred to females during copulation (Bartelt et al. 1985). This pheromone can attract flies over distance. At closer distance, the flies could rely on visual cues but also on chemicals sensed by sensory neurons on the ovipositor (Chess & Ringo 1985), the proboscis, wings, and legs to help them decide whether to lay an egg (Chyb 2004; Meunier et al. 2000). Additional tests would be needed to determine if the order in which cues are detected could have an effect on the oviposition outcome.

Previous studies of larval aggregation have shown that *D. melanogaster* exhibited a greater degree of aggregation than *D. simulans* (Durisko et al. 2014). They proposed that one of the benefits of group borrowing is that it facilitates larval penetration of food to hide from parasitoid wasps. Interestingly, *D. melanogaster* and *D. simulans* have different defense strategies against parasitoids with *D. melanogaster* exhibiting specific avoidance behavior against parasitoid wasps (Ebrahim et al. 2015), while *D. simulans* exhibits greater physiological immune responses (Lefevre et al. 2012). It could be that the greater larval sociality observed among *D. melanogaster* larvae serves to increase their burrowing ability as a strategy to reduce parasitism. In ovipositing adult females, this could also be one of the factors that explains why we saw significant preference to oviposit together with conspecifics in all treatments tested synchronously (Fig. 1C), and significant aggregation of conspecific females in oviposition sites, even in darkness (Fig. 2).

In conclusion, our findings provide new insights into the oviposition behavior of *D. melanogaster* and *D. simulans*, revealing a preference for social oviposition, even in an interspecific context. This indicates that the benefits of social oviposition, in many cases, outweigh the risks of competition. This aligns with the principles of the Allee effect, where smaller, isolated populations face greater challenges in fitness and survival due to difficulties in cooperation and resource acquisition (Courchamp et al. 1999). The results also suggest that chemical cues and social interactions both could play a role in shaping these oviposition choices. Future studies could explore the ecological consequences of such social strategies in natural populations, as well as the mechanisms underlying these behaviors. Understanding these dynamics broadens our understanding of cooperation, competition, and resource use in species that share overlapping ecological niches.

## Methods

### Fly stocks

The study utilized wild-type flies that were acquired from the National Drosophila Species Stock Centre (NDSSC; http://blogs.cornell.edu/drosophila/) and the Kyoto stock center (Kyoto DGGR; https://kyotofly.kit.jp/cgi-bin/stocks/index.cgi): *D. melanogaster* (Hansson’s lab) and *D. simulans* flies (Stock no. *14021‐0251.01*). We also used mutant flies acquired from the Bloomington Drosophila Stock Center (BDSC; https://bdsc.indiana.edu/): *D. melanogaster* that expresses GFP ubiquitously under control of Act5C (RRID:BDSC_4534), and *D. melanogaster* with an *Orco* gene mutation (RRID:BDSC_23130). All flies were raised under specific conditions: a temperature of 25 °C, a 12-h light and 12-h dark cycle, and 70% relative humidity. The flies were reared at standard density in vials with standard cornmeal diet, with males and females together. Per liter of diet, it consists of 118 g of beet syrup, 11 g of brewer's yeast, 95 g of yellow cornmeal, 4.1 g of agar (Carl Roth ®), 2.4 ml of Propionic acid (> 99% pure, 13.4 M; Carl Roth ®) and 3.3 ml of 30% Nipagin (Sigma-Aldrich ®). The care and treatment of all flies adhered to applicable ethical regulations.

### Chemical Analyses

#### Thermal Desorption-Gas Chromatography-Mass Spectrometry (TD-GC–MS)

In order to compare the chemical profiles of the mutant *D. melanogaster* mated females (RRID:BDSC_4534) with the wild type flies, newly hatched females were left with males to mate, which does not exclude multiple matings. Individual 10-day old flies were decapitated to avoid them from escaping. They were placed in standard microvials in thermal desorption tubes and transferred into a GERSTEL thermal desorption unit (www.gerstel.de) using a GERSTEL MPS 2 XL multipurpose sampler. We analyzed at least 6 replicates. All the cuticular chemical profiles from wild type flies (16 species) were generated in a previous study (Moreira-Soto et al. 2024) following the same procedure.

Regarding the GC–MS system, an Agilent GC 7890 A coupled with an MS 5975 C inert XL MSD unit (www.agilent.com) was utilized, featuring an HP5-MS UI column (19091S-433UI; Agilent Technologies). Volatiles were initially desorbed at 250 °C for 8 min, then trapped at − 50 °C by means of liquid nitrogen cooling. To introduce the components into the GC column, the vaporizer injector was progressively heated to 270 °C at a rate of 12 °C per second and maintained at that temperature for 5 min. The GC oven temperature was held at 50 °C for 3 min, then increased at a rate of 15 °C per minute to 250 °C, where it was held for 3 min, before being further increased to 280 °C at a rate of 20 °C per minute, and maintained for 20 min. For the MS, the transfer line, source, and quadrupole were kept at 270 °C, 230 °C, and 150 °C, respectively.

The raw GC/MS data were converted to AIA format using MSD ChemStation (Agilent Technologies). These converted files were then imported into R (version 4.1.0), where the XCMS package was employed for peak detection and retention time alignment (Smith et al. 2006). For peak detection in XCMS, the centWave algorithm was applied with the following settings: ∆*m/z* tolerance of 30 ppm, minimum peak width of 3 s, maximum peak width of 50 s, and a signal-to-noise threshold of 20. Retention time correction was achieved using the obiwarp function, and peaks were grouped with parameters of an *m/z* width of 0.1, a base width of 5, and a minimum fraction of 0.1. All chromatographic peaks occurring before 540 s and after 1980 s were excluded. This analysis was done to compare the chemical profiles of mutant *D. melanogaster* mated females, along the chemical profiles of 16 *Drosophila* species, including *D. simulans* and *D. melanogaster,* taken from a previous study (Moreira-Soto et al. 2024).

The XCMS data (intensities of compounds, i.e., features with distinct *m/z* (mass-to-charge ratios)) was normalized by the sum of all features per sample. From this, samples were compared using a Uniform Manifold Approximation and Projection (UMAP) in R (4.1.0) with umap package (McInnes et al. 2020), using the default values for the parameters (n_neighbors = 15, min_dist = 0.1). We tested for statistical difference in the abundance from all compounds found, with Tukey’s test for multiple comparisons of means (P < 0.05), using GraphPad Prism v. 9 (https://www.graphpad.com).

### Behavioral Experiments

#### Oviposition Assays

All behavioral experiments were conducted throughout multiple months, with the different test situations being mixed, to avoid that they were run with different cohorts of flies. Tests were carried out to determine whether a single *D. melanogaster* fly would oviposit together or separately with a single conspecific or, in another set of experiments, with a *D. simulans* fly when multiple oviposition sites were available. In order to distinguish eggs from two different *D. melanogaster* females or from one *D. melanogaster* and one *D. simulans* female, we used a *D. melanogaster* fly line (RRID:BDSC_4534) expressing GFP ubiquitously under control of Act5C. This mutant fly line was used for all assays where 2 flies were tested together (Fig. 1 C_i-iv_ and D_i-iv_). We tested these mutant *D. melanogaster* flies against *D. simulans*, or wild type *D. melanogaster* as control, using 7–10 days old female flies. These flies were separated by sex upon hatching and maintained in groups in vials with fly food. The day before the assays, males and females were placed together (in group) in vials containing 5% sucrose and yeast powder for 24 h before the assays began.

In cubic cages of 45 cm per side, we set up the oviposition assays for 24 h, where flies had 4 equal small petri dishes (diameter, 3.5 cm) containing fly food, in several different conditions. Afterwards, the eggs on each petri dish were counted (assays that did not yield in any oviposition were excluded from further analyses). Using individual flies, we also tested several different conditions that potentially would give the flies less reasons to cooperate: we either used half the amount of agar in the fly food (see recipe above) to soften the substrate or enriched the oviposition substrate with 20 µl of a 2% yeast solution. On the other hand, we tested increasing competition by making the oviposition surface smaller, using Eppendorf tubes (diameter 1 cm) filled with fly food as oviposition sites. For the treatments, the number of replicates was between 15 and 26 (see Supplementary Data 1). All behavioral experiments were performed under normal white light at 25 °C and 70% humidity.

To test the oviposition of *D. simulans* with *D. melanogaster*, or 2 flies of the latter species, in a sequential manner, we set up the same 4 choice oviposition assay using petri dishes as described before, but one fly was left 24 h to oviposit, and was then removed before adding the other fly. In this case, eggs were counted after the first fly had the opportunity to oviposit, and then counted again after the second fly oviposited. This approach also allowed us to use only wild type flies for the sequential assays, as the eggs were counted separately, making fluorescent labeling unnecessary.

All the experiments were conducted in blocks, with each treatment tested across multiple months. To assess the preference for oviposition, we only used the data of the positive assays, i.e. assays in which both flies laid eggs (see Table S2). From these positive assays we calculated the Spearman’s Rank Correlation Coefficient using the function cor in the stats R package 4.1.0, where a value close to 1 means that they lay eggs on the same place, and values close to −1 where the eggs were laid separately. For example, if one fly lays 7 eggs on plate A and the other fly lays 15 eggs on the same plate A, we reach a Spearman correlation of 1. If one fly lays 18 eggs on plate A, and the other fly lays 25 eggs on plate A, and 3 eggs on plate B, the Spearman correlation drops to 0.82. On the other hand, if they lay eggs separately, where one fly lays 20 eggs on plate A, and the other lays 7 eggs on plate B and 2 eggs on plate C, we obtain a Spearman correlation of −0.54. To reach a Spearman correlation of −1, both flies need to lay the same number of eggs, distributed equally in 2 plates for each fly; for example, one fly lays 10 eggs in plate A and 10 in plate B, and the other fly lays 10 in plate C and 10 in plate D. To statistically test if the correlations were significantly different from zero, Wilcoxon signed-rank tests were conducted using GraphPad Prism v. 9 (https://www.graphpad.com).

#### Aggregation Assays

In order to test, whether females are influenced by the presence or absence of other females when they target an oviposition site, we used a similar setup as before, but instead of oviposition plates now installed 4 traps. We tested 5 *D. melanogaster* flies against 5 conspecifics or 5 *D. simulans*, using 7–10 days old female flies mated the day before the assays, and left in vials with 5% saccharose and yeast powder until the assays started. In cubic cages of 45 cm per side, we set up the trap assays for 24 h, where flies had 4 equal vials with fly food (diameter, 3.5 cm) provided with a pipette tip as a one-way entrance, i.e. where they could get in but not come out (Fig. 2A). Experiments were performed under normal white light at 25 °C and 70% humidity. To test the role of vision on this behavior we repeated the tests with no light. In order to test the role of olfaction in the behavior, we used *D. melanogaster* flies with a mutation rendering their olfactory coreceptor Orco non-functional (RRID:BDSC_23130).

Flies on each of the traps were counted, and separated morphologically by species in the case of mixed assays, using descriptions from McEvey (2019). In order to assess the aggregation, we calculated Morisita’s standardized aggregation index using the vegan R package (Oksanen et al. 2022). Values closer to 1 indicate a significant aggregation, while values closer to 0 indicate a random distribution, and values closer to −1 indicate a uniform distribution of flies in the traps. For example, if 9 flies are found in 1 trap, and the other 3 traps are empty, we reach a standardized Morisita’s index of 1. If we find 5 flies on trap A, 2 flies on traps B and C, and 1 fly on trap D, the standardized Morisita’s index calculated is 0.05. If we find 1 fly in each of the 4 traps, we reach a standardized Morisita’s index of −1. To statistically test if the indexes were significantly different from zero, Wilcoxon signed-rank tests were conducted using GraphPad Prism v. 9 (https://www.graphpad.com).

## Supplementary Information

Below is the link to the electronic supplementary material.

Supplementary file1 (XLSX 30 KB)

Supplementary file2 (XLSX 11 KB)

Supplementary file3 (XLSX 11 KB)

Supplementary file4 (PDF 168 KB)

## Data Availability

All the raw data and other relevant data supporting the findings of this study are provided as supplementary files.
