# Supplementary figures and images for: Oviposition Dynamics and Niche Utilization in Two Sympatric Drosophila Species
Source: J Chem Ecol. 2025 Feb 4;51(1):21. doi: 10.1007/s10886-025-01576-4 (PMC11794365; doi:10.1007/s10886-025-01576-4)

UMAP plot

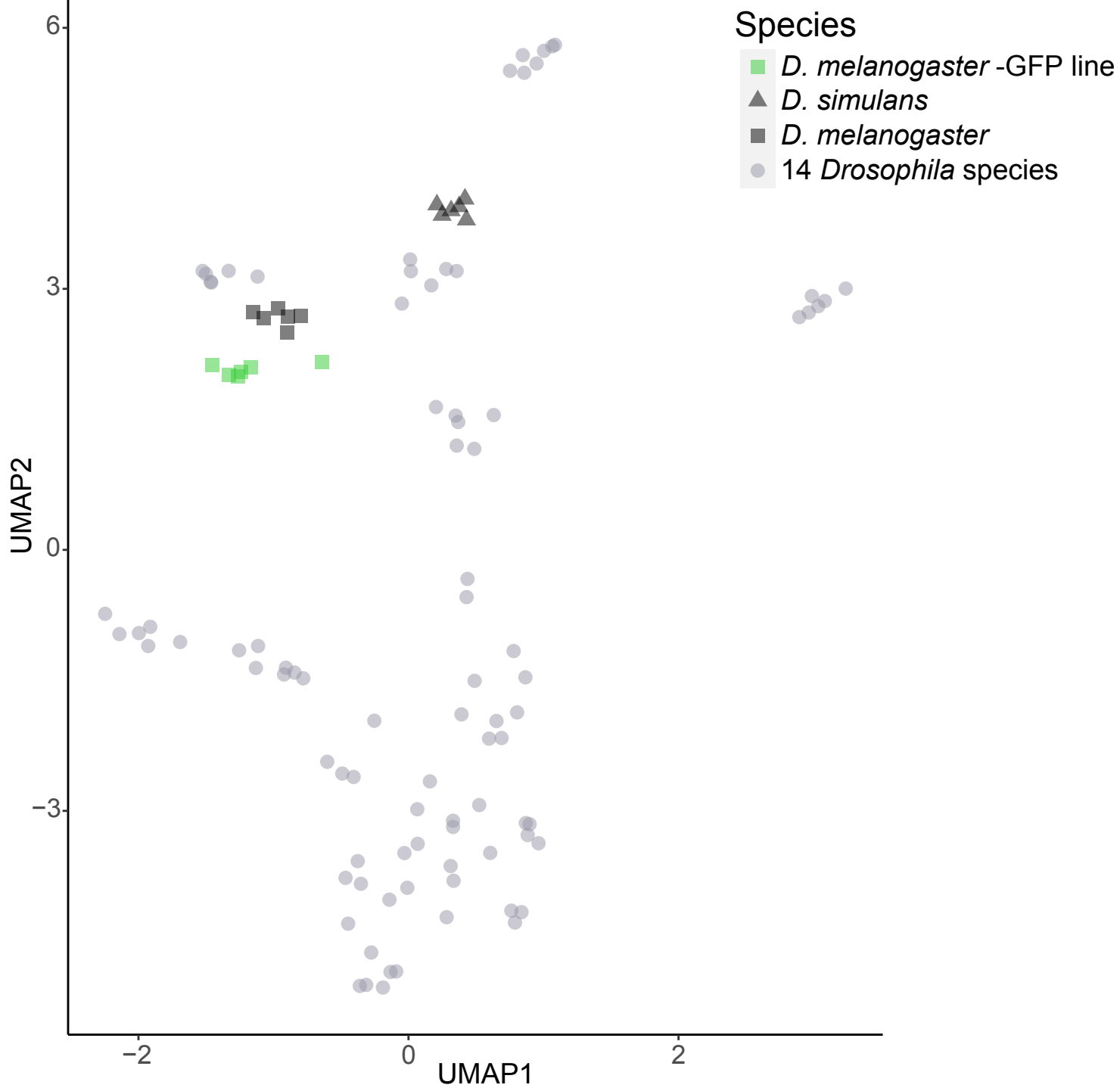

Supplement: Supplementary file 4 — Supplementary file4 (PDF 168 KB) [file 10886_2025_1576_MOESM4_ESM.pdf]
